# Discerning best practices in XFEL-based biological crystallography – standards for nonstandard experiments

**DOI:** 10.1107/S205225252100467X

**Published:** 2021-06-30

**Authors:** Alexander Gorel, Ilme Schlichting, Thomas R. M. Barends

**Affiliations:** a Department of Biomolecular Mechanisms, Max Planck Institute for Medical Research, Jahnstr. 29, Heidelberg, 69120, Germany

**Keywords:** serial femtosecond crystallography, data analysis, X-ray free-electron lasers, error models, time-resolved crystallography, extrapolated structure-factor amplitudes, structural biology

## Abstract

Serial femtosecond crystallography (SFX) at X-ray free-electron lasers (XFELs) is a novel tool in structural biology. In contrast to conventional crystallography, SFX relies on merging partial intensities acquired with X-ray beams of often randomly fluctuating properties from a very large number of still diffraction images of generally randomly oriented microcrystals. For this reason, and possibly due to limitations of the still evolving data-analysis programs, XFEL-derived SFX data are typically of a lower quality than ‘standard’ crystallographic data. In contrast with this, the studies performed at XFELs often aim to investigate issues that require precise high-resolution data, for example to determine structures of intermediates at low occupancy, which often display very small conformational changes. This is a potentially dangerous combination and underscores the need for a critical evaluation of procedures including data-quality standards in XFEL-based structural biology. Here, such concerns are addressed.

## Abstract 2

Serial femtosecond crystallography is coming of age. Therefore, it is time to consider best practices and appropriate standards.

## 1. Crystallography at X-ray free-electron lasers   

X-ray free-electron lasers (XFELs) have become a transform­ative force in structural biology. Their extreme peak brilliance and unique time structure have enabled studies hitherto believed impossible, such as structure determination of metalloenzymes considered free of radiation damage (Suga *et al.*, 2015 ▸; Hirata *et al.*, 2014 ▸), characterization of protein dynamics with sub-picosecond time resolution (Barends, Foucar *et al.*, 2015 ▸; Pande *et al.*, 2016 ▸; Coquelle *et al.*, 2018 ▸; Nogly *et al.*, 2018 ▸; Nass Kovacs *et al.*, 2019 ▸; Skopintsev *et al.*, 2020 ▸) and X-ray data collection from *in vivo* grown nanocrystals (Colletier *et al.*, 2016 ▸). The general enthusiasm that has accompanied these success stories is evident from the commentaries written about the various high-profile publications describing these investigations. Detailed reviews on advances in XFEL-based structural biology have been published [see *e.g.* Schlichting (2015*a* ▸), Spence (2017 ▸) and Chapman (2019 ▸)] as well as on the various approaches to efficiently deliver crystals into the XFEL beam (Grünbein & Kovacs, 2019 ▸; Martiel *et al.*, 2019 ▸).

Continuous advances in synchrotron beamline capabilities and the development of ever-better detectors and data-processing software have brought macromolecular crystallography to a point where high-precision data can often be collected from comparatively small crystals with minimal effort. At XFELs, the situation is very different. XFEL-based macromolecular crystallography typically uses a method named serial femtosecond crystallography (SFX) (Chapman *et al.*, 2011 ▸; Boutet *et al.*, 2012 ▸). Because XFEL pulses are extremely intense, every X-ray exposure using a focused XFEL beam typically destroys the sample. Therefore, a new crystal (or fresh region thereof) is required for every diffraction image, and these are introduced into the beam on a solid support or, more often, in a liquid column. Thus, nonisomorphism between crystals, as well as variations in crystal size and quality, result in large variations between measurements. Added to this are fluctuations in beam profile, beam pointing, beam intensity and even beam spectrum, which at XFELs vary strongly from shot to shot. Moreover, because XFEL pulses have durations of femtoseconds, the crystals cannot be rotated during exposure as is the case in ‘conventional’ crystallography. This means that only ‘still’ images are recorded, so reflection intensities are only partially measured. In SFX, these problems are solved by averaging over a large number of exposures (Monte Carlo integration), which, given sufficient observations, converges to the correct relative magnitudes of the reflection intensities (Kirian *et al.*, 2011 ▸). Indeed, there is a clear correlation between the number of images and the precision of the data as indicated by CC_1/2_, CC* and the value of *R*_split_, an *R* factor related to *R*_p.i.m._ adapted to Monte Carlo integration (White *et al.*, 2012 ▸; Barends *et al.*, 2014 ▸; Gorel *et al.*, 2017 ▸). However, in practice, beam time at XFELs is extremely competitive and the collection of hundreds of thousands of images is not typically feasible, even if sufficient sample is available. In addition, there is the necessarily experimental nature of the detectors at XFEL beamlines. They are faced with the tremendous task of integrating a signal that lasts only femtoseconds in a high-radiation environment and then rapidly communicating that signal to a storage buffer. Enormous advances have been made here (Strüder *et al.*, 2010 ▸; Philipp *et al.*, 2010 ▸; Blaj *et al.*, 2015 ▸; Henrich *et al.*, 2011 ▸; Kuster *et al.*, 2014 ▸; Allahgholi *et al.*, 2019 ▸; Hatsui *et al.*, 2014 ▸; Kameshima *et al.*, 2014 ▸; Redford *et al.*, 2018 ▸) but it is still likely that systematic errors are introduced into the final intensities. To a large extent, this is due to the limited dynamic range these detectors currently have or to problems caused by the automatic gain switching methods employed to extend that range. Considering everything, it is no surprise that XFEL-derived macromolecular-crystallography data are not yet of the quality the crystallographic community at large has become accustomed to: for instance, the overall signal-to-noise ratio 〈*I*/σ(*I*)〉 of such a dataset is typically lower than 5–6, much lower than the typical values for synchrotron datasets.

Every new field, at its conception, is faced with a problem: while there is a need for standards of good practice as for every other field, there are usually insufficient data points and a lack of experience or even understanding of some underlying principles to base such standards on. This, however, also applies to fields that have suddenly made a quantum leap in capabilities: consider, for instance, cryo-electron microscopy, which has existed as a field for decades but which is now faced with redefining standards because of the current ‘resolution revolution’ (Lyumkis, 2019 ▸; Lawson *et al.*, 2020 ▸). As XFEL-based crystallography is maturing, the community is now in a good position to define such standards, particularly as these standards can be based on synchrotron-based structural biology. Here we aim to provide a list of topics we believe need to be considered when defining standards for XFEL-based structural biology, starting from the existing standards for ‘conventional’ macromolecular crystallography.

## 2. Processing SFX data – status, challenges   

To a traditional crystallographer it seems inconceivable that experimental parameters such as crystal-to-detector distance, X-ray wavelength and properties of the X-ray detector are not known precisely. However, currently this is the typical situation for SFX at XFELs. The experimental setups are often rebuilt between different experiments, which may explain the large uncertainties associated with the assumed detector distances and the lack of precise geometry information of tiled detectors. Detector calibrations are often compromised by drifting or changing offsets, hot pixels, the need for frequently updated dark file corrections, incorrect gain settings, *etc*. These issues can affect both the magnitude of the extracted diffraction intensities as well as the accuracy and ease of indexing. Indexing of diffraction data of macromolecular crystals obtained by rotation approaches is greatly helped by the constraint that any initial guess concerning the crystal lattice symmetry and orientation of the lattice in the X-ray beam must be consistent with the indexing of subsequent diffraction patterns with increased total rotation angle. This advantage is missing in SFX unless one uses very large crystals for XFEL data collection that can be exposed at consecutive fresh locations differing by rotational increments of Δφ and stepwise translations of Δ*x* and Δ*y* [guided by experimentally determined mosaicity and damage-zone values, respectively (Hirata *et al.*, 2014 ▸; Cohen *et al.*, 2014 ▸)], an approach dubbed serial femtosecond rotation crystallography (Schlichting, 2015*b* ▸). In contrast, when using microcrystals for data collection, the diffraction data are collected serially with a fresh randomly oriented crystal for each exposure, resulting in a still diffraction image. Indexing such patterns is not only more difficult but also less accurate than indexing a set of rotation images. Indeed, processing a large set of finely sliced rotation images either in series (rotation method, *XDS*) or independently (randomly drawn single images, *nXDS*), Kabsch observed significantly worse statistics for the latter approach (Kabsch, 2014 ▸).

At present, SFX data processing is mainly performed with one of two software packages, *CrystFEL* (White *et al.*, 2012 ▸) and *cctbx.xfel* (Sauter *et al.*, 2013 ▸; Hattne *et al.*, 2014 ▸). Surprisingly, no benchmarking as described above for *nXDS* has been published. As with the detectors, sample-delivery systems, *etc.*, the ongoing development of these programs has been essential to the continuing success of XFEL-based structural biology. Although the philosophy underlying these two software packages differs in some crucial aspects, systematic comparisons of their performance are unexpectedly scarce. For a brand new approach such as SFX, in which novel programs are used, this is regrettable, all the more so as datasets to base such a comparison on are readily available. One of only two systematic comparisons (Sawaya *et al.*, 2014 ▸) observed no relevant differences in final structures produced from either *CrystFEL*- or *cctbx.xfel*-derived data; however, the authors did note a striking difference in the Wilson *B* factor, which was considerably lower for the *cctb.xfel*-derived data. This in turn affected the electron-density maps, particularly for *e.g.* water molecules; the net effect of using *cctbx.xfel* rather than *CrystFEL* being an effective sharpening of the map. The apparent cause of this is the difference in treating weak high-resolution data: whereas *cctbx.xfel* determines a maximum resolution for each image, *CrystFEL* normally uses the same resolution for the entire dataset, resulting in the inclusion of weak or even zero intensities from images collected from weakly diffracting crystals. This would also explain the large difference in *I*/σ(*I*) reported by the other study comparing the two programs (Hattne *et al.*, 2014 ▸). The resulting difference in the maps’ ‘sharpness’ noted by Sawaya *et al.* may not be important for the determination of the overall fold of a protein, but becomes very relevant indeed when small structural changes, possible at partial occupancies, are to be studied, as is the case in many high-impact XFEL studies. In our experience, for such studies it is therefore essential that investigators carefully evaluate (and show) the intensity statistics of their data, using *e.g.* the Wilson plot and the *N*(*Z*) plots provided by programs such as *phenix.xtriage* (Adams *et al.*, 2010 ▸) or *TRUNCATE* (Winn *et al.*, 2011 ▸). This also helps to decide on the effective resolution of the data (see also Section 2.1).

A few approaches towards improving the quality of SFX data by accounting for the partiality of individual measurements have been described, such as the method reported by Kabsch in *nXDS* (Kabsch, 2014 ▸) which calculates the offset from each reciprocal lattice point to the Ewald sphere to correct for the partiality of the individual measurements. A post-refinement method was added by White to the *CrystFEL* software package (White, 2014 ▸) but at the time was only tested on simulated data. Working with thermolysin data measured at an XFEL, Sauter described a partiality model that resulted in clear improvement of data statistics (Sauter, 2015 ▸). Ginn and coworkers also described an improvement of their XFEL data quality upon inclusion of a partiality model (Ginn, Messerschmidt *et al.*, 2015 ▸; Ginn, Brewster *et al.*, 2015 ▸) but did so using virtually perfect crystals with a mosaicity of 0.03° and with strong high-resolution data, where the limit was imposed by the detector size.

As already mentioned, SFX data are generally of lower quality than ‘traditional’ rotation data. In addition to the lack of 3D profile fitting (Kabsch, 2014 ▸), a number of issues may contribute, including stochastic shot-to-shot variations in crystal quality, XFEL pulse energy (intensity) as well as X-ray photon energy and even spectral distribution. Intuitively, one would think that the influence of the latter would contribute strongly to accurate indexing and thus ultimately data quality. However, comparisons of the quality of SFX data acquired using either a self-amplified spontaneous emission (SASE) or seeded (quasi monochromatic) XFEL beam showed none (Barends, White *et al.*, 2015 ▸) or no major differences (Nam *et al.*, 2021 ▸). Along these lines, serial synchrotron crystallography (SSX) data collected analogously as SFX data, see Diederichs & Wang (2017 ▸) for a review, but using single-shot (micro to) millisecond exposures of monochromatic X-rays, are of similar quality to XFEL-acquired SFX data (Mehrabi *et al.*, 2021 ▸). Nevertheless, to allow successful *de novo* phasing, the SSX data contained far fewer images (Botha *et al.*, 2015 ▸) than typically required for SFX data (Barends *et al.*, 2014 ▸). This could be due to the fact that appreciable rotation of jet-delivered crystals is possible during the relatively long SSX exposure times (Botha *et al.*, 2015 ▸), effectively resulting in quasi-rotation images.

### 2.1. Judging data quality   

The infamous ‘Table 1’, listing data and refinement statistics, has been, for better or worse, a staple of macromolecular-crystallography articles. Among other things, this table reports various data-quality metrics that ideally should help the reader judge whether the data support the conclusions of the study. As such, it is often one of the first things to look at, even before reading the main text of an article. However, it has become increasingly recognized that the usefulness of many metrics typically reported in ‘Table 1’ is limited, and an excellent case was made recently (Rupp, 2018 ▸) that a graphical display of quality metrics as a function of position in reciprocal space is much more informative. Nevertheless, the reporting of some metrics remains an accepted standard. Chief among these are various statistics informing on signal strength and the precision of the data, such as *I*/σ(*I*), *R*_merge_ or *R*_p.i.m._, CC_1/2_ and CC*, see Karplus & Diederichs (2015 ▸) for a review. These are usually reported both for the entire resolution range as well as for the highest resolution shell of the data. Indeed, these metrics are typically used to decide on the resolution to which the data are considered useful and to which they are to be used. Thus, the data are cut at some resolution where *e.g.* *I*/σ(*I*), CC_1/2_ and CC* are still acceptably high, and/or where *R*_merge_ or *R*_p.i.m._ are still acceptably low. What is deemed acceptable has shifted over the years towards including ‘weaker’ data, afforded by better detectors and driven by the development of refinement strategies employing Bayesian statistics that take such data into account in a mathematically rigorous way.

SFX data-processing software such as the *CrystFEL* suite are able to provide reports on several data-quality metrics. Several of these indicate the consistency between two ‘half datasets’: the individual observations are divided into two equal parts and two sets of structure factors are calculated from them. These are then used to calculate an *R* factor between them such as *R*_split_, or the correlation coefficient CC_1/2_. *R*_split_, like *R*_merge_, *R*_p.i.m._ and other *R* factors, increases with resolution, and when little data can be collected may be extremely high in the highest resolution shell, sometimes exceeding 100%. This would appear to indicate that these data contain little to no information, but in such cases other metrics, such as CC_1/2_, often indicate that even at high resolution there is still some correlation between the two half datasets, indicating information content. As for synchrotron-based crystallography, CC_1/2_ is often used to calculate CC*, an estimate of the correlation between the measured intensities and their true values (‘CC_true_’) (Karplus & Diederichs, 2015 ▸).

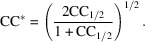

**Figure 1:**
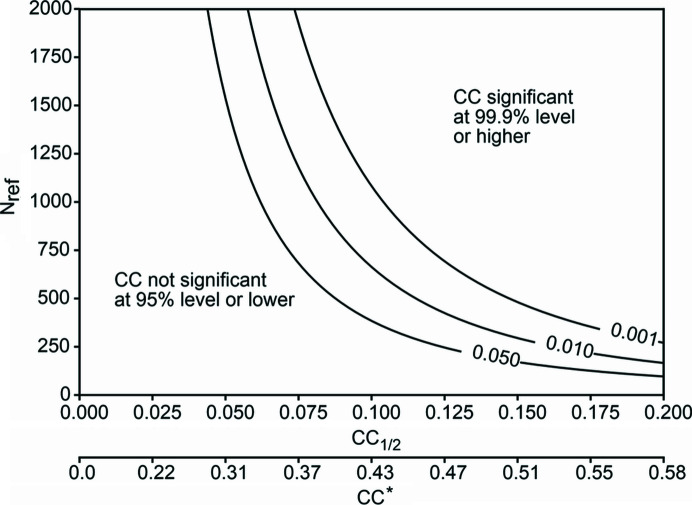
A contour plot showing the probability α that a certain CC_1/2_ value calculated for a particular number of observations *N*_ref_ arises from pure chance. The lines for α = 0.05, 0.01 and 0.001 are plotted. Below the α = 0.05 line, the correlation would not be considered significant and thus is produced by noise at 95% level of confidence or even lower. This may correspond to an appreciable value for CC* (lower axis).

Particularly at low values for CC_1/2_, this can suggest a surprisingly high information content despite extremely high values of *R*_split_. Indeed, a recent article (Kern *et al.*, 2014 ▸) reports XFEL data being used to a resolution where CC_1/2_ = 0.4%, which, while apparently low, would translate to a CC* of ∼9%. However, in contrast to synchrotron data statistics, comparing intensities of fully integrated Bragg reflections, XFEL data statistics compare merged intensity values of random slices through reciprocal lattice points. The latter only converge to the expected Bragg intensity in the case of a very large number of crystal hits (multiplicity of measurements of the partial intensities). This can be challenging for high-resolution reflections. Thus, when using correlations, one needs to consider the possibility that a certain correlation arises from mere chance. Fig. 1 ▸ shows the probability of this occurring for a certain CC_1/2_ and number of observations *N*_ref_ (see the supporting information for a derivation). Although low values of CC_1/2_ correspond to relatively high values of CC*, when a shell contains comparatively few reflections, the probability of CC_1/2_ suggesting a correlation where none actually exists becomes appreciable. Indeed, even with >24 000 unique observations in the highest resolution shell as in the above-mentioned case, the probability that the reported CC_1/2_ has arisen by chance is as high as 0.53. For this reason, *XDS* indicates whether a correlation coefficient is significant at the 0.1% level by performing a T-test.

A better indication of the resolution at which the data still contain information may be obtained by ‘paired refinement’ (Diederichs & Karplus, 2013 ▸): comparing model *R* factors and electron-density maps obtained of successive refinement runs with stepwise inclusion of reflections of higher resolution shells as was carried out manually for SFX data by Bublitz *et al.* (2015 ▸). The ‘paired refinement’ protocol including a cross-validation procedure has been automated recently (Malý *et al.*, 2020 ▸). However, using a single (‘spherical’) resolution cut-off may be inappropriate in cases where the data are anisotropic or where the data-collection method makes them appear so. In SFX, this latter scenario may occur when the injection process results in some crystal orientations being preferred over others. This can happen when *e.g.* gas-dynamic virtual nozzles are used to inject needle-shaped crystals that align along their long axis when passing through the nozzle. In such cases, it may be more appropriate to use an ellipsoidal resolution cut-off as implemented in *STARANISO* (Vonrhein *et al.*, 2018 ▸).

In conclusion, much more than in conventional crystallography one needs to look at various statistical data qualifier metrics together, and inspect the resolution dependence of Wilson and *N*(*Z*) plots. Ultimately one needs to check the electron-density maps to judge the information contents of the data. Nice-looking maps, however, may of course be misleading, particularly with SFX data which can be weaker than a traditional crystallographer is used to. Thus, even more than in conventional synchrotron-based crystallography, it is good practice to test for model bias by omitting the coordinates of a large residue or cofactor from the model and to check whether the density comes back in the map. This is particularly important when judging maps calculated from extrapolated structure-factor amplitudes (see Section 3.2).

Many SFX experiments have goals beyond a mere static structure determination, such as testing the influence of certain experimental parameters on data quality or the characterization of short-lived reaction intermediates in time-resolved experiments. Similar to the data statistics listed in Table 1, knowledge of the critical parameters of the experiment (*e.g.* pulse energy, pulse length, pump-laser parameters, …) quickly helps the reader judge whether the data and the experimental design can support the conclusions of the study. However, typically the values of the respective experimental parameters are contained somewhere in material and methods sections or in supporting information preventing fast assessment. Thus, it would be very useful to either add such parameters to Table 1 (for example X-ray pulse energy and peak spectral intensity when comparing SASE and seeded beam data) or list them in an additional Table (see Table 1 ▸ for a pump probe experiment. Analogous tables can be made for chemical mixing experiments, listing flow speed, concentrations, …).

## 3. Challenging structure determinations   

### 3.1. *De novo* phasing   

Given the lower quality of SFX data it was initially doubtful whether such data would be good enough for *de novo* phasing. After the first proof-of-principle demonstration, which used the huge anomalous signal of a Gd derivative for single-wavelength anomalous-diffraction phasing of lysozyme (Barends *et al.*, 2014 ▸), basically every single phasing approach has been demonstrated using model systems (Yamashita *et al.*, 2015 ▸; Nakane *et al.*, 2016 ▸; Nass *et al.*, 2016 ▸, 2020 ▸; Hunter *et al.*, 2016 ▸; Batyuk *et al.*, 2016 ▸; Gorel *et al.*, 2017 ▸). Given that the amount of protein required for SFX experiments is much larger than for conventional macromolecular crystallography and in view of the challenges often associated with growing appropriate crystals for SFX, it is perhaps not surprising that there is only one previously unknown structure (BinAB) that was determined by *de novo* phasing using SFX data (Colletier *et al.*, 2016 ▸). BinAB crystallizes in nanocrystalline form *in vivo*; since the crystals are too small for data collection using synchrotron radiation, solving the structure by SFX was the only way forward. This required a major effort involving purification of nanocrystals and their subsequent derivatization with heavy atoms, the ‘blind’ collection of >300 000 indexable diffraction images (*i.e.* without the benefit of on-line analysis capabilities such as fluorescence scans), and processing the data with novel experimental software followed by a highly convoluted phasing process requiring the optimization of many parameters.

### 3.2. Low-occupancy intermediates   

The high peak brilliance and unique time structure of XFEL radiation enables time-resolved crystallographic studies – by facilitating efficient reaction initiation – at unprecedented temporal and spatial resolution. Although we focus on light-induced reactions, most aspects addressed in the following deal with the analysis of reaction intermediates of typically low occupancy, which is relevant to time-resolved studies in general. The earliest of several ultrafast pump probe SFX experiments examined ligand dissociation of carb­oxy-myoglobin (Barends, Foucar *et al.*, 2015 ▸), which can be triggered by visible light with essentially unity quantum yield. Importantly, the intensity of the femtosecond light pulses must be limited and crystals smaller than the penetration depth of the photoexciting light must be used to prevent multiphoton excitation and to achieve high occupancy of reaction intermediates, respectively (Grünbein *et al.*, 2020 ▸). It is noteworthy that in the single photon excitation regime for two-level systems less than 50% activation is possible even with 100% quantum yield. However, most systems have a far lower photoexcitation quantum yield. This results in the reaction intermediates of interest being present in the crystals at medium to low occupancies at best (Pande *et al.*, 2016 ▸; Coquelle *et al.*, 2018 ▸; Nogly *et al.*, 2018 ▸; Nass Kovacs *et al.*, 2019 ▸; Skopintsev *et al.*, 2020 ▸).

To assess structural light-induced changes in molecules induced by X-ray crystallography, an approach he coined photocrystallography [see Coppens (2003 ▸)], Philip Coppens introduced the RATIO method (Coppens *et al.*, 2009 ▸). This approach is based on back-to-back diffraction images acquired with (ON) and without (OFF) a laser pump pulse. By merging *I*_ON_/*I*_OFF_ ratios of integrated spot intensities for each indexed reflection, instead of intensity differences, errors arising from image scaling and wavelength normalization (when using polychromatic X-rays) are avoided. Therefore, the RATIO method has also been used successfully for time-resolved pump probe studies on macromolecules by Laue crystallography (Schotte *et al.*, 2012 ▸). Thus, it would seem that the RATIO method should be well suited for the analysis of time-resolved pump probe SFX data acquired with a monochromated beam (avoiding shot-to-shot changes in spectral composition of the SASE beam which would fail to cancel out). However, when we analysed pairs of laser-ON and laser-OFF diffraction images of large single myoglobin crystals collected at the LCLS we observed non-systematic large changes, preventing use of the RATIO method. These could have been caused by shot-to-shot changes in XFEL beam pointing – and possibly other beam parameters (Loh *et al.*, 2013 ▸) – resulting not only in different parts of the crystal being probed but also in significant changes in background scattering.

An alternative to the RATIO method is to assess reaction-induced changes in diffraction intensities by analysing integrated intensities. The first task to be performed is to calculate difference electron densities between the reference dataset (laser-OFF in the case of light-triggered reaction) and the dataset acquired at a certain time delay after reaction triggering. This provides fast feedback on the magnitude of reaction-induced changes. The differences depend on the occupancy of the reaction intermediate(s) as well as the extent of the structural changes. While the absence of any difference electron-density peaks, in particular at longer time delays after triggering (where structural changes are expected to be large), suggests failure of the experiment, not all conformational differences manifest as difference peaks and may only become apparent after refinement.

Refining a model of a partially occupied state is, however, a problem for protein crystallography, even with high-quality synchrotron data, because of the typical resolution limits in macromolecular crystallography and the concomitant low observation/parameter ratios. At intermediate occupancies, perhaps 20% or higher, ensemble refinement may be able to deal with the situation. At lower occupancies, ‘extrapolated structure factors’ or rather extrapolated structure-factor amplitudes are typically calculated, which approximate the structure-factor amplitudes of the excited fraction of the crystal by extrapolating to 100% occupancy, assuming zero phase difference between ground- and excited-state structure factors (Genick, 2007 ▸; Genick *et al.*, 1997 ▸).

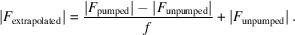

Here, |*F*_pumped_| and |*F*_unpumped_| are the amplitudes recorded from the photoexcited and ‘dark’ crystals, respectively, and *f* is the expected occupancy of the reaction intermediate. The resulting positive and negative values of |*F*_extrapolated_| may then be used to calculate a map or serve as a refinement target.

The calculation of such amplitudes raises some issues however, particularly when XFEL data are used, since the typically large errors in such data propagate into very large errors for the extrapolated amplitudes, particularly at low values of *f* (Genick, 2007 ▸). This results in noisy maps that are difficult to interpret and noisy amplitudes that are difficult to refine against. Moreover, the result depends crucially on the value of *f*, which is typically determined by inspecting the density. Two ways of estimating *f* from the map can currently be found in the XFEL literature. One is to visually inspect the map starting with a low value for *f*, increasing it until signs of the unpumped state start to appear in the density (Nogly *et al.*, 2018 ▸; Nass Kovacs *et al.*, 2019 ▸; Weinert *et al.*, 2019 ▸). Though based on human interpretation, this method chiefly relies on features of the unpumped state, which is a known structure. This approach would therefore appear to be relatively ‘safe’ in that little or no bias towards prior expectations is introduced. Other authors (Pande *et al.*, 2016 ▸), however, vary *f* while automatically comparing a map calculated from the expected structural change with the extrapolated map until the differences are minimized. However, the results of this approach (with values of *f* reported with an unrealistic precision of three decimal places) should be treated with caution, as this method obviously relies on prior expectations about the pumped structure. In all likelihood, for all current studies, there is a large uncertainty in *f* which in turn increases the error bars on any results derived from the data.

Another point of disagreement in the community, surprisingly, appears to be the choice of the unpumped dataset. The safest experimental approach is to collect pumped and unpumped data in an interleaved fashion. This helps address systematic errors such as non-isomorphism between different crystal batches used for data collection, shifts in photon energy, changes in focus, *etc*. However, this approach is often not used and different schools of thought appear to exist concerning the reference data. One school uses the actually measured unpumped amplitudes and their sigmas (Nass Kovacs *et al.*, 2019 ▸), whereas another uses data calculated from a refined unpumped structure (Pande *et al.*, 2016 ▸). This latter approach might be problematic, as any errors in the unpumped structural model would be propagated into the extrapolated amplitudes, such as coordinate errors but also *e.g.* inadequacies in modelling the bulk solvent. Moreover, when calculated structure factors are used for the unpumped structure, no error estimates for *F*_unpumped_ are available to estimate the errors in the final extrapolated amplitudes. Some studies (Nogly *et al.*, 2018 ▸; Weinert *et al.*, 2019 ▸) report simply substituting σ*F*_pumped_ for σ*F*_extrapolated_, which would result in a dramatic and unwarranted underestimation of the true errors, in turn causing an overestimation of the signal-to-noise ratio of the data, and consequently of its resolution.

Related to this issue is the treatment of negative values of |*F*_extrapolated_|. These arise from the assumptions underlying the extrapolation in combination with errors in the observed amplitudes, which exacerbates the problem in the case of XFEL-derived data. Some authors delete these from their datasets (Nogly *et al.*, 2018 ▸; Weinert *et al.*, 2019 ▸), likely improving *R* factors, whereas others retain them (Nass Kovacs *et al.*, 2019 ▸). While no systematic study of this particular subject has been reported yet, experience with negative intensities in structure refinement has shown that retaining such values, when an appropriate refinement target is used, is preferable (Read & McCoy, 2016 ▸), as even just recognizing that an intensity or amplitude is small adds a restraint to the refinement, helping to improve model quality. Moreover, removing negative amplitudes will mostly remove reflections that have been strongly affected by the unpumped → pumped transition. This may therefore affect more than just the estimation of the occupancy of the pumped state determined by inspecting electron-density maps calculated from extrapolated structure-factor amplitudes.

Finally, there is the issue of which phases to use when inspecting (or building into) extrapolated maps. It would appear to be prudent to use phases calculated from the unpumped state, but Pande *et al.* (2016 ▸) used an iterative mixing scheme in which, after calculating extrapolated structure-factor amplitudes based on a refined model for the dark state, they calculated phases from models of the ground and excited states and used these to perform structure-factor extrapolation in the complex plane. This then resulted in new extrapolated amplitudes, against which a new model for the excited state was refined *etc.* This would appear to risk introducing phase bias through the back door, which may result in the changes caused by photoexcitation to be overestimated.

#### 3.2.1. Validating time-resolved series   

Often, structural changes in response to a trigger are investigated at a series of time delays between trigger and data collection so as to obtain a time series or ‘molecular movie’. When the time points are spaced sufficiently close, it is reasonable to assume that the magnitudes of the changes in electron density and thus of the structural changes vary more or less smoothly with time. This can be leveraged to help distinguish between signal and noise: spurious peaks that occur only at one time point are more likely to be caused by noise than features varying continuously over several time delays. Moreover, it appears equally reasonable to assume that *e.g.* light-triggered structural changes will mainly be present close to the chromophore at short time delays, spreading out over the rest of the protein only at later times. This, too, can be used as a consideration when estimating the noise level of difference maps. Several tools exist that aid such analyses. Wickstrand *et al.* (2020 ▸) describe a method to represent time-dependent changes in electron-density maps in a 1D way, by separately averaging negative and positive difference density values within a pre-defined radius around certain atoms. The resulting plots allow time-dependent trends in density changes to be spotted very conveniently. Alternatively, a time series of (difference) electron-density maps may be analysed by cluster analysis (Kostov & Moffat, 2011 ▸) or singular value decomposition (Schmidt *et al.*, 2010 ▸; Nass Kovacs *et al.*, 2019 ▸). The latter separates the data into orthogonal components, each with its own development over time. In favourable cases, this may even allow some noise to be removed from the maps by excluding components that do not vary smoothly with time.

#### 3.2.2. Error estimates for small structural changes   

Typically, the structural changes reported in XFEL-based ultrafast time-resolved crystallographic studies are of the order of a few tenths of Ångstroms (Barends, Foucar *et al.*, 2015 ▸; Nass Kovacs *et al.*, 2019 ▸; Skopintsev *et al.*, 2020 ▸). This is close to the typical average coordinate precision in (synchrotron) macromolecular crystallography and, given the concerns about XFEL data quality discussed above, there is a clear need to validate such results. However, generally, the resolution limit of the data does not allow full-matrix refinement, so variances of individual coordinates cannot be obtained directly.

**Figure 2:**
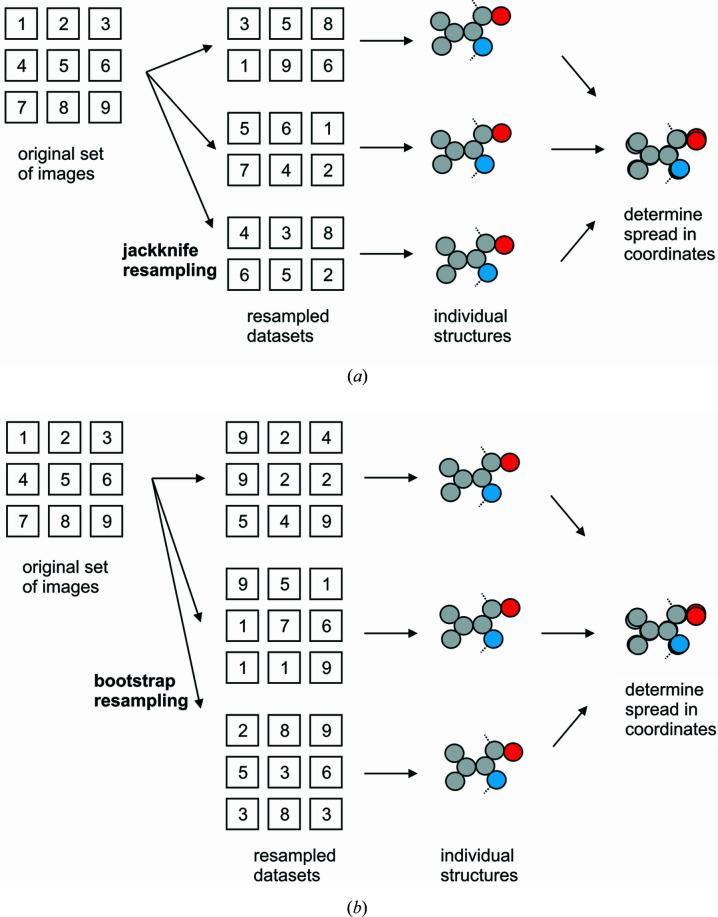
Resampling can be used to estimate the effect of measurement errors in SFX data on final refined coordinates. (*a*) Jackknifing-type resampling, see Nass Kovacs *et al.* (2019 ▸). Multiple resampled datasets are constructed by randomly drawing images from the entire original pool of diffraction images, resulting in resampled datasets that are smaller (70–90%) than the original dataset. Structures are determined from each of these, which are then compared to obtain an estimate of the variation in the atomic positions. (*b*) Bootstrap [see Grünbein, Foucar *et al.* (2021 ▸)] resampling is similar to jackknifing, but the resampling is performed by ‘random drawing with replacement’, which means that after random selection of an image from the pool a copy is placed in the resampled dataset and the original image is put back in the original pool. In this way, multiple resampled datasets are constructed that contain the same number of images as the original pool but in which images can be represented multiple times.

A practicable way to obtain estimates of coordinate errors is a resampling approach such as ‘jackknifing’ (Fig. 2 ▸). This entails refining multiple structures, each against a dataset integrated from a randomly selected subset of the available indexed SFX images. One then compares the resulting structures to obtain an estimate of how much the coordinates depend on the variation in the individual observations (Nass Kovacs *et al.*, 2019 ▸). This approach is, however, in principle, sensitive to the size of the subsets; smaller subsets may be expected to yield larger variations. It may therefore be more appropriate to use the closely related ‘bootstrapping’ method (Fig. 2 ▸). This method, too, relies on independently refining structures against multiple resampled datasets, but here those resampled datasets contain the same number of images as the original dataset and are obtained by ‘random drawing with replacement’, *i.e.* after selecting an image for inclusion in the resampled set it is placed back into the pool. In this way, each image can in principle be placed in the same resampled set multiple times (Grünbein, Gorel *et al.*, 2021 ▸).

The computational cost of these resampling methods may be prohibitive, in particular during a beam time when a quick answer is required to guide data-collection efforts. In such a case, the ‘gold standard’ half-dataset method used in cryo-electron microscopy may be employed to obtain a feeling for the coordinate errors, by splitting the available images into two separate datasets and independently refining two models against them, after which the resulting structures are compared, as was carried out by Barends, Foucar *et al.* (2015 ▸).

## 4. Deposition of XFEL data   

**Figure 3:**
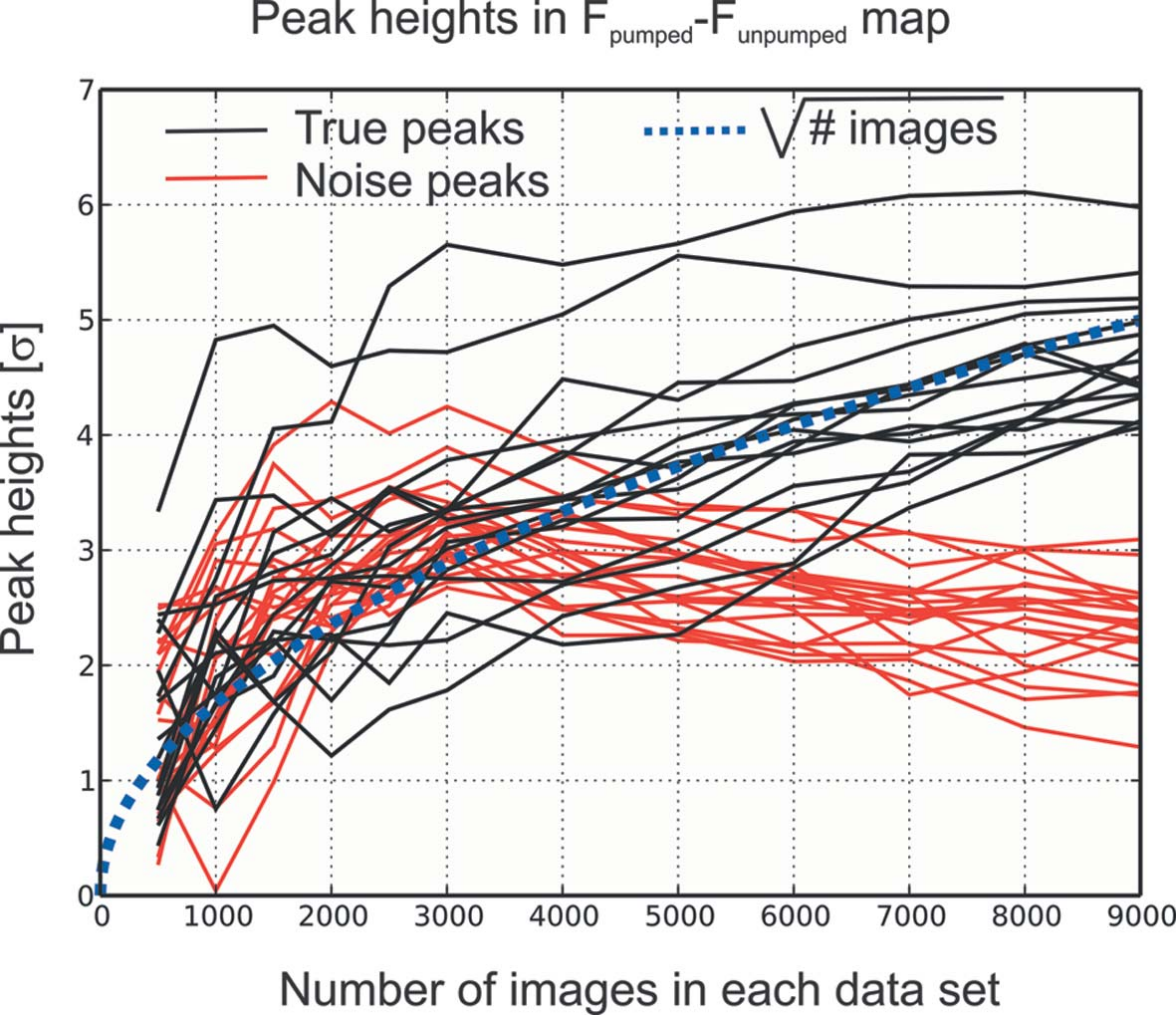
The development of peak heights in an |*F*_pumped_| − |*F*_unpumped_| difference map derived from SFX data as a function of the number of images. The data used are from bacteriorhodopsin, 33 ms after light excitation (Nass Kovacs *et al.*, 2019 ▸), to a resolution of 1.8 Å. Using the maps calculated with 3000 and 9000 images in both datasets, 15 ‘real’ peaks caused by structural change on light excitation were chosen, as well as 18 ‘false’ (spurious) peaks that could not be distinguished from the true signal when 3000 images were used for each dataset. Both positive and negative peaks were selected, and the negative peaks inverted so as to have positive values. The heights of these peaks (black: true peaks, red: false peaks) were plotted as a function of the number of images used for both datasets. The dashed blue line is proportional to the square root of the number of images.

Protein crystallography has a long-standing tradition of requiring the deposition of structures before publication, and for many years now the deposition of structure-factor amplitudes has been required by journals too. This enables readers to judge whether the conclusions of a crystallographic article are backed up by the data. Indeed, various ways now exist that allow convenient retrieval of all that is needed for the calculation of final refined (2*mF*_o_ − *DF*_c_ and *mF*_o_ − *DF*_c_) electron-density maps, or, with a little bit more work, various OMIT maps. This is an undoubted strength that crystallography shares with few other fields and which deserves careful preservation (Bernstein *et al.*, 2020 ▸). The XFEL community, however, has produced articles that violate the norm (Kupitz *et al.*, 2017 ▸), or at least the spirit of the norm (Pande *et al.*, 2016 ▸; Nogly *et al.*, 2018 ▸), by discussing in great detail electron-density maps for which no data was deposited, as no structure was refined. Thus, it is impossible for readers to judge the quality of these maps for themselves, or to calculate different ones to analyse the influence of the procedure (see above) on the outcome of the map and thus on the mechanistic interpretation. This is especially problematic with XFEL data given the comparatively low qualities of typical SFX datasets, as shown in Fig. 3 ▸. Here, the development of peak heights in an |*F*_pumped_| − |*F*_unpumped_| difference map derived from SFX data is shown as a function of the number of images, both for ‘real’ peaks that are due to the structural change under investigation as well as for ‘false’ (spurious) peaks. Until the point where as many as 9000 images (the total number of images that was collected for the pumped dataset) are used to determine both |*F*_pumped_| and |*F*_unpumped_|, there is little or no difference in peak height between the true and false peaks, despite the fact that the example chosen concerns a large structural change [bacteriorhodopsin 33 ms after light excitation (Nass Kovacs *et al.*, 2019 ▸)] and that the resolution of the data was 1.8 Å. As expected for Monte Carlo integrated data, the ‘real’ peaks grow in height approximately proportional to the square root of the number of images, whereas the spurious peaks first grow and then slowly taper off in height. Thus, even if twice as much data could have been collected in the available time, the signal-to-noise ratio would not have increased dramatically. Given the scarcity of XFEL beam time and the difficulty of making sufficient sample that is often encountered, this presents a huge problem for experiments of this kind. Indeed, observations reported in a high-impact publication (Kupitz *et al.*, 2014 ▸) have already been hotly debated (Sauter *et al.*, 2016 ▸), largely because of the high noise level of the difference maps that were presented (a discussion that was only possible because the diffraction data had been deposited). Thus, in our opinion, journals should require deposition of data and map coefficients even when no structure is produced from them; in the end, a structure is just an interpretation, whereas map coefficients allow the merits of that interpretation to be evaluated, and data even allow the calculation of the various types of maps to be repeated by the reader. As the Protein Data Bank (PDB) does not currently allow the deposition of stand-alone datasets or map coefficients, such results could be added to a article as supporting information. This would for instance be useful in the case of extrapolated structure factors, which the PDB sometimes accepts for deposition and sometimes does not (Tobias Weinert and Jacques-Philippe Colletier, personal communications), or when special weighting schemes such as *q* weighting (Terwilliger & Berendzen, 1995 ▸; Ursby & Bourgeois, 1997 ▸) have been used to calculate difference-map coefficients (Coquelle *et al.*, 2018 ▸). A flexible format for the deposition of different types of map coefficients in the PDB might thus be of great help in preserving the spirit of the norm mentioned above and carrying it on into the future. Alternatively, datasets can be deposited as raw images in the Coherent X-ray Imaging Data Bank (CXIDB) (Maia, 2012 ▸), which has the added advantage of allowing them to be used in the development of data-processing software.

## 5. Conclusions   

During the last decade, XFEL-driven macromolecular crystallography has emerged as a novel approach in structural biology producing exciting and important results that are beyond the reach of synchrotron-based crystallography or electron microscopy. While coming of age, SFX is not yet as mature as synchrotron-based crystallography. Partly driven by the rapidly evolving technical possibilities, and the ensuing need for new analysis tools, SFX has appropriated traditional crystallographic approaches, often however without proper benchmarking or controls. Thus, there is a danger of a discrepancy between what an XFEL-based study aims to do and what it is actually able to deliver in terms of data quality. As our experience with SFX data increases, our community must come up with best-practice approaches, and ways to correctly assess the quality of SFX-derived results to see which conclusions can be drawn from them and which cannot. Now is the time to do this, as enough experience, data and different analysis tools are at hand for systematic comparisons. Conveniently, the vast body of experience with conventional crystallography in terms of *e.g.* quality indicators can be used as a starting point. However, most data-quality indicators try to reduce a very complicated story to a few numbers. We believe none of these, and least of all the resolution, are really useful in judging whether the inferences drawn from a typical XFEL study are justified. In our opinion, the insight gained from statistical quality markers is even more limited for SFX data than for synchrotron data so it is essential to carefully evaluate the electron-density maps and, when structural changes are being studied, the error estimates on the final coordinate using resampling methods. Moreover, to aid identifying best practices and thus advancing and maturing the still young and evolving field, all data on which interpretations are based, and where necessary and practicable, should not only be made available but reassessed comparatively.

## Supplementary Material

Statistical assessment of correlations. DOI: 10.1107/S205225252100467X/be5289sup1.pdf

## Figures and Tables

**Table 1:** Parameters for pump probe experiments

		Comment
Laser parameters	Pulse energy (µJ)	
	Beam profile	Gaussian, top hat
	Beam diameter (µm)	Indicate FWHM or 1/*e* ^2^
	Pulse duration (fs)	
	Spatial offset of laser and X-ray foci (µm)	
	Pump wavelength λ_P_ (nm)	
	Laser polarization	
	Laser fluence (mJ cm^−2^)†	
	Laser power (GW cm^−2^)†	
Sample parameters	Extinction coefficient at λ_P_ (*M* ^−1^ cm^−1^)	
	Average sample thickness (µm)‡	
	Unit-cell parameters: *a*, *b*, *c*, α, β, γ (Å,°)	
	Number of chromophores per unit cell	
	Chromophore concentration (m*M*)	Derived quantity for crystals
	λ_P_ extinction length (µm)†	Calculated quantity
Photoexcitation	Nominal number of absorbed photons per chromophore†	

† These cells indicate data derived from the experimental values. They provide fast feedback on the photoexcitation regime and need to be listed somewhere in the manuscript. It makes sense to add them to this table. The extinction length of the pump laser light has direct implications for the design of the experiment.

‡ For the calculation of an average path length through crystals see page 9 of the supporting information of Nass Kovacs *et al.* (2019 ▸).
