# Supplementary material for: Discerning best practices in XFEL-based biological crystallography – standards for nonstandard experiments
Source: IUCrJ. 2021 Jun 30;8(Pt 4):532–43. doi: 10.1107/S205225252100467X (PMC8256713; doi:10.1107/S205225252100467X)
Supplement: Supplementary file 1 [file m-08-00532-sup1.pdf]

IUCrJ

Volume 8 (2021)

Supporting information for article:

Discerning best practices in XFEL-based biological crystallography – standards for nonstandard experiments

Alexander Gorel, Ilme Schlichting and Thomas R. M. Barends

S1. Calculation of the correlation between the two sets of fixed number of random and independent variables

For the calculation of $CC_{1/2}$, the entire dataset of images is split into two equal parts. Both halves are processed independently to arrive at two separate sets of intensities. The correlation between these two half-datasets is calculated in resolution shells, to give a $CC_{1/2}$ for each shell. The values of $CC_{1/2}$ are then used to help define a resolution cut-off and the data are truncated at a resolution where $CC_{1/2}$ falls below an arbitrary value. The expectation is that for strong, accurately determined data, the two half-datasets will show a strong correlation, whereas for noise, the correlation will be weak. However, it is important to put numbers on this expectation. Thus, we need to know the probability that a certain $CC_{1/2}$ arises from pure noise. In other words, we need to quantify the significance level of any calculated $CC_{1/2}$ value.

In the following we derive a formula for the probability of a nonzero correlation from pure coincidence. To this end, we employ a noise model for the integrated intensities that allows the calculation of a significance level. To obtain a significant value for $CC_{1/2}$ and thus to define a resolution cut-off it is required that the calculated correlation value exceeds the correlation arising from random coincidence.

The correlation of noise can be understood as a random variable Z that is calculated from the covariance of two random variables X and Y, which are the individual intensities in the two half datasets:

$$Z = Corr[X,Y] = \frac{Cov[X,Y]}{\sigma_x \sigma_y}$$

Where the covariance is defined as

$$Cov[X,Y] = \sum_{i=1,\dots,N} p\,[x_i, y_i](x_i - \mu_x)(y_i - \mu_y)$$

With the assumption that the integrated intensities from the background noise x_i and y_i are identically and independently distributed, this results in the definition of the random variable Z as follows:

$$Z = \frac{1}{N} \sum_{i=1,\dots,N} \left(\frac{x_i - \mu_i}{\sigma_x}\right)\left(\frac{y_i - \mu_y}{\sigma_y}\right)$$

In the following, we derive the distribution of the correlation value as a random variable and the parameters of this distribution using the algebra of random variables.

$Z \propto ?\,[\mu_Z, \sigma_Z]$

The individual x_i and y_i values are calculated as the mean values of the integrated intensities for the identical Miller indices $\widetilde{x_i}$ and $\widetilde{y_i}$.

$$x_i = \frac{1}{M} \sum_{j=1,\dots,M} \tilde{x}_\iota$$

These values display a normal, *i.e.* Gaussian distribution, since the Central Limit Theorem states that the sum of identically and independently distributed random variables, that need not themselves be normal distributed, approaches a normal distribution with a new mean and new standard deviation for the case that the number of summands approaches infinity; *i.e.:*

$$x_i \propto Gauss[\mu_{x_i}, \sigma_{x_i}]$$

The mean intensities are calculated as the mean of a set of intensity measurements, *i.e.* the normalized sum of random variables. Hence, for a large number of observations of an intensity, the probability distribution of the mean value can be approximated by a normal distribution with appropriate mean and standard deviation parameters.

Since, given a large number of observations, we can apply the Central Limit Theorem to the approximation of the probability distribution of the mean intensity values, we are free to choose the probability distribution for the individual values. If we assume that these values $\tilde{x}_J$ are produced by uncorrelated noise, we can choose the probability distribution to simply be uniform on the interval of [0,1] for the individual measurements $\tilde{x}_J$.

$$\tilde{x}_J \propto Uniform[\{0,1\}] = Uniform\left[\tilde{\mu}_J = 0.5, \tilde{\sigma}_J = \frac{1}{12}\right]$$

According to the Central Limit Theorem, the probability distribution for the sum of many random variables is Gaussian with new mean and standard deviation parameters, where $\tilde{\mu}$ is the mean of the individual measurements, and $\tilde{\sigma}$ their standard deviation.

$$\tilde{X}_\iota = \sum_{j=1,\dots,M} \tilde{x}_\iota$$

$$\mu_{\tilde{X}_\iota} \rightarrow \sum_{j=1,\dots,M} \tilde{\mu}_J = M \cdot \tilde{\mu}$$

$$\sigma_{\tilde{X}_\iota} \rightarrow \sqrt{\sum_{j=1,\dots,M} \tilde{\sigma}_J^{\,2}} = \sqrt{M} \cdot \tilde{\sigma}$$

$$\tilde{X}_\iota \propto Gauss[\mu_{\tilde{X}_\iota} = M\tilde{\mu}, \sigma_{\tilde{X}_\iota} = \sqrt{M} \cdot \tilde{\sigma}]$$

Scaling this random variable changes the mean and the standard deviation but not the distribution.

$$x_i = \frac{1}{M} \tilde{X}_\iota$$

$$\mu_{x_i} \rightarrow \frac{1}{M} \cdot \mu_{\tilde{X}_\iota}$$

$$\sigma_{x_i} \rightarrow \frac{1}{M} \cdot \sigma_{\tilde{X}_\iota}$$

$$x_i \propto Gauss\left[\mu_{x_i} = \tilde{\mu}, \sigma_{x_i} = \frac{\tilde{\sigma}}{\sqrt{M}}\right]$$

Shifting and scaling the random variable again changes the mean and standard deviation of the distribution to zero mean and a standard deviation of 1.

$$x_i - \mu_{x_i} \propto Gauss[0, \sigma_{x_i}]$$

$$X_i = \frac{x_i - \mu_{x_i}}{\sigma_{x_i}} \propto Gauss[0,1]$$

Now the correlation value random variable Z can be understood as the sum of products of the individual observables for the different mean integrated reflection intensities.

$$Z = Corr[X, Y] = \frac{1}{N} \sum_{i=1,\dots,N} X_i \cdot Y_i$$

Multiplying two standard Gaussian-distributed random variables produces a random variable that has a different probability distribution. With X_i and Y_i being gaussian distributed the Z_i will follow a Bessel-K distribution. (The derivation of the probability distribution of a product of two Gaussian-distributed random variables is shown further below).

$$X_i \propto Gauss[0, \sigma_{X_i}]$$

$$Y_i \propto Gauss[0, \sigma_{Y_i}]$$

$$Z_i = X_i \cdot Y_i \propto PDF_{Z_i}[Z_i; \sigma_{X_i}, \sigma_{Y_i}] = \frac{1}{\pi \sigma_{X_i} \sigma_{Y_i}} BesselK\left[0, \frac{|Z_i|}{\sigma_{X_i} \sigma_{Y_i}}\right]$$

The mean of this distribution is 0 and the standard deviation for Z_i amounts to 1, as will be shown below using the characteristic function.

$$\mu_{Z_i} = 0$$

$$\sigma_{Z_i} = \sqrt{\sigma_{X_i}^2 \sigma_{Y_i}^2} = 1$$

$$Z_i \propto PDF_{Z_i}[Z_i; \sigma_{X_i} = 1, \sigma_{Y_i} = 1] = \frac{1}{\pi} BesselK[0, |Z_i|]$$

The sum of many random variables that are Bessel-K distributed can be again approximated by a normal distributed random variable according to the Central Limit Theorem.

$$Z = Corr[X, Y] = \frac{1}{N} \sum_{i=1,,N} Z_i$$

The summation of Z_i produces ζ which is normally distributed.

$$\zeta = \sum_{i=1,,N} Z_i$$

$$\zeta \propto Gauss[0, \sqrt{N}]$$

Z can now be calculated by scaling the ζ random variable, and its (Gaussian) probability density function can now be determined:

$$Z = Corr[X,Y] = \frac{1}{N}\zeta$$

$$Z = Corr[X,Y] \propto Gauss\left[\mu_Z = 0, \sigma_Z = \frac{1}{\sqrt{N}}\right]$$

By integrating the probability density function of Z, a general formula for the significance value of $CC_{1/2}$ given the number of reflection intensities N in a resolution shell and the significance level α (0.05,0.01,0.001 ...) can be derived:

$$(1-\alpha) = \int_{-CC_{1/2}^{sig}}^{CC_{1/2}^{sig}} PDF_Z\,[Z;\mu_Z,\sigma_Z]dZ = \frac{1}{2}\left(Erf\left[\frac{CC_{1/2}^{sig} - \mu_Z}{\sqrt{2}\sigma_Z}\right] + Erf\left[\frac{CC_{1/2}^{sig} + \mu_Z}{\sqrt{2}\sigma_Z}\right]\right)$$

Using the previously derived values for the distribution statistics

$\mu_Z = 0$ and

$\sigma_Z = 1/\sqrt{N}$

it follows that

$(1-\alpha) = Erf\left[\frac{CC_{1/2}\cdot\sqrt{N}}{\sqrt{2}}\right]$ and

$$CC_{1/2}^{sig} = \frac{\sqrt{2}InverseErf[(1-\alpha)]}{\sqrt{N}}$$

The required number N^{sig} of structure factors in a resolution shell for a significant correlation given a $CC_{1/2}^{sig}$ value can be calculated from the following formula.

$$N^{sig} = \frac{2\cdot InverseErf[(1-\alpha)]^2}{\left(CC_{1/2}^{sig}\right)^2}$$

These formulae can be used to calculate whether an obtained $CC_{1/2}$ given the number of intensities in the given resolution shell is statistically significant.

S2. Calculation of the PDF for the product of two Gaussian random variables

In this section, we show how to obtain the probability distribution for the product of two random variables using the algebra of random variables.

The probability density function (PDF) is defined as the derivative of the cumulative density function (CDF).

$$PDF_Z[z] = \frac{d}{dz}(CDF_Z[z])$$

For the case that the random variable Z is defined as the product of two random variables X and Y, the CDF is defined as follows:

$$CDF_Z[z] = \int_{-\infty}^{\infty}\int_{-\infty}^{\infty} PDF_X\,[x]PDF_Y[y]\Theta[z-(x\cdot y)]dxdy$$

where Θ is the Heaviside step function.

Using the Fourier transform and the inverse Fourier transform

$$F[f,x\to\omega] = \frac{1}{\sqrt{2\pi}}\int_{-\infty}^{\infty} f\,[x]exp[-i\omega x]dx$$

$$F^{-1}[g,\omega\to x] = \frac{1}{\sqrt{2\pi}}\int_{-\infty}^{\infty} g\,[\omega]exp[i\omega x]d\omega$$

$$F^{-1}[F[f,x\to\omega],\omega\to x] = f[x]$$

and the linearity property of the Fourier transform

$$F[a\cdot f+b\cdot h,x\to\omega] = a\cdot F[f,x\to\omega]+b\cdot F[h,x\to\omega]$$

we can perform this integration:

$$CDF_Z[z] = \int_{-\infty}^{\infty}\int_{-\infty}^{\infty} PDF_X\,[x]PDF_Y[y]F^{-1}[F[\Theta[z-(x\cdot y)],z\to\omega],\omega\to z]dxdy$$

$$CDF_Z[z] = F^{-1}\left[\int_{-\infty}^{\infty}\int_{-\infty}^{\infty} PDF_X\,[x]PDF_Y[y]F[\Theta[z-(x\cdot y)],z\to\omega]dxdy,\omega\to z\right]$$

The Fourier transform of the unit step function Θ is obtained as follows:

$$F[\Theta[z-(x\cdot y)],z\to\omega] = \frac{-i}{\sqrt{2\pi}\omega}exp[-i\omega xy]$$

Finally, to calculate the PDF for the product of two Gaussian-distributed random variables with their respective PDFs

$$PDF_X[x] = \frac{exp\left[\frac{-y^2}{2\sigma_x^2}\right]}{\sqrt{2\pi}\sigma_x}$$

$$PDF_Y[y] = \frac{exp\left[\frac{-y^2}{2\sigma_y^2}\right]}{\sqrt{2\pi}\sigma_y}$$

we insert these definitions into the equation

$$CDF_Z[z] = F^{-1}\left[\int_{-\infty}^{\infty}\int_{-\infty}^{\infty} \frac{exp\left[\frac{-x^2}{2\sigma_x^2}\right]}{\sqrt{2\pi}\sigma_x} \frac{exp\left[\frac{-y^2}{2\sigma_y^2}\right]}{\sqrt{2\pi}\sigma_y} \left(\frac{-i}{\sqrt{2\pi}} exp[-i\omega xy]\right) dxdy, \omega \to z\right]$$

and obtain the following integral after integration and application of the definition of the inverse Fourier transform given above. This integral does not have a tabulated solution.

$$CDF_Z[z] = \frac{1}{\sqrt{2\pi}}\int_{-\infty}^{\infty} \frac{-i}{\sqrt{2\pi}\omega\sqrt{1+\omega^2\sigma_x^2\sigma_y^2}} exp[i\omega z]d\omega$$

Differentiation can be performed within the integral to obtain the probability density function:

$$PDF_Z[z] = \frac{d}{dz}(CDF_Z[z])$$

$$= \frac{d}{dz}\left(\frac{1}{\sqrt{2\pi}}\int_{-\infty}^{\infty} \frac{-i}{\sqrt{2\pi}\omega\sqrt{1+\omega^2\sigma_x^2\sigma_y^2}} exp[i\omega z]d\omega\right)$$

$$= \frac{1}{\sqrt{2\pi}}\int_{-\infty}^{\infty} \frac{-i}{\sqrt{2\pi}\omega\sqrt{1+\omega^2\sigma_x^2\sigma_y^2}} \frac{d}{dz}(exp[i\omega z])d\omega$$

$$= \frac{1}{\sqrt{2\pi}}\int_{-\infty}^{\infty} \frac{-i}{\sqrt{2\pi}\omega\sqrt{1+\omega^2\sigma_x^2\sigma_y^2}} i\omega exp[i\omega z]d\omega$$

$$= \frac{1}{\sqrt{2\pi}}\int_{-\infty}^{\infty} \frac{1}{\sqrt{2\pi}\sqrt{1+\omega^2\sigma_x^2\sigma_y^2}} exp[i\omega z]d\omega$$

$$= \frac{1}{\pi\sigma_x\sigma_y} BesselK\left[0, \frac{|z|}{\sigma_x\sigma_y}\right]$$

S3. Calculating the mean and standard deviation of a Bessel-K-distributed random variable using the characteristic function

In the following, we show how the statistics (mean and standard deviation) for an arbitrary probability distribution can be obtained using the characteristic function of the probability distribution.

The characteristic function is defined as follows:

$$C_Z[\omega] = E\big[exp[i\omega z]\big] = \int_{-\infty}^{\infty} PDF_Z\,[z] exp[i\omega z] dz$$

For the case of the Bessel-K distribution this produces:

$$C_Z[\omega] = \frac{1}{\sqrt{1+\omega^2\sigma_x^2\sigma_y^2}}$$

The n-th moment is defined as:

$$m_n = \int_{-\infty}^{\infty} PDF_Z\,[z] \cdot z^n dz$$

Which is related to the characteristic function by:

$$m_n = (-i)^n \frac{d^n}{d\omega^n}(C_Z[\omega])_{\omega=0} = (-i)^n \frac{d^n}{d\omega^n}\left(\frac{1}{\sqrt{1+\omega^2\sigma_x^2\sigma_y^2}}\right)_{\omega=0}$$

The first moment for the Bessel-K distribution can now be derived using:

$$m_1 = (-i)\left(\frac{-\omega\sigma_x^2\sigma_y^2}{1+\omega^2\sigma_x^2\sigma_y^2}\right)_{\omega=0} = 0$$

And the second moment can be derived using this relation.

$$m_2 = (-i)^2\left(\frac{3\omega^2\sigma_x^4\sigma_y^4}{\left(1+\omega^2\sigma_x^2\sigma_y^2\right)^{\frac{5}{2}}} - \frac{\sigma_x^2\sigma_y^2}{\left(1+\omega^2\sigma_x^2\sigma_y^2\right)^{\frac{3}{2}}}\right)_{\omega=0} = \sigma_x^2\sigma_y^2$$

The variance and therefore the standard deviation (as the square root of the variance) can be derived from the difference of the 2nd and (squared) 1st moments:

$$var_{Z_i} = m_2 - (m_1)^2 = \sigma_x^2\sigma_y^2$$

$$\sigma_{Z_i} = \sqrt{\sigma_x^2\sigma_y^2}$$
